# Structural and functional features of self-assembling protein nanoparticles produced in endotoxin-free *Escherichia coli*

**DOI:** 10.1186/s12934-016-0457-z

**Published:** 2016-04-08

**Authors:** Fabián Rueda, María Virtudes Céspedes, Alejandro Sánchez-Chardi, Joaquin Seras-Franzoso, Mireia Pesarrodona, Neus Ferrer-Miralles, Esther Vázquez, Ursula Rinas, Ugutz Unzueta, Uwe Mamat, Ramón Mangues, Elena García-Fruitós, Antonio Villaverde

**Affiliations:** Institut de Biotecnologia i de Biomedicina, Universitat Autònoma de Barcelona, Bellaterra, Cerdanyola del Vallès, 08193 Barcelona, Spain; Departament de Genètica i de Microbiologia, Universitat Autònoma de Barcelona, Bellaterra, Cerdanyola del Vallès, 08193 Barcelona, Spain; CIBER de Bioingeniería, Biomateriales y Nanomedicina (CIBER-BBN), Bellaterra, Cerdanyola del Vallès, 08193 Barcelona, Spain; Biomedical Research Institute Sant Pau (IIB-SantPau) and Josep Carreras Leukemia Research Institute, Hospital de la Santa Creu i Sant Pau, 08025 Barcelona, Spain; Servei de Microscòpia, Universitat Autònoma de Barcelona, Bellaterra, Cerdanyola del Vallès, 08193 Barcelona, Spain; Leibniz University of Hannover, Technical Chemistry & Life Science, Hannover, Germany; Helmholtz Centre for Infection Research, Inhoffenstraße 7, Brunswick, Germany; Division of Structural Biochemistry, Priority Area Asthma and Allergy, Research Center Borstel, Airway Research Center North (ARCN), Member of the German Center for Lung Research (DZL), 23845 Borstel, Germany; Cibbim-Nanomedicine, Hospital Vall d’Hebron, Passeig de la Vall d’Hebron, 119-129, 08035 Barcelona, Spain; Department of Ruminant Production, Institut de Recerca i Tecnologia Agroalimentàries (IRTA), Torre Marimon, Caldes de Montbui, 08140 Barcelona, Spain

**Keywords:** Protein engineering, Recombinant proteins, Nanoparticles, Nanomedicine, Biomaterials, Biodistribution, *E. coli*, Endotoxin-free strains

## Abstract

**Background:**

Production of recombinant drugs in process-friendly endotoxin-free bacterial factories targets to a lessened complexity of the purification process combined with minimized biological hazards during product application. The development of nanostructured recombinant materials in innovative nanomedical activities expands such a need beyond plain functional polypeptides to complex protein assemblies. While *Escherichia coli* has been recently modified for the production of endotoxin-free proteins, no data has been so far recorded regarding how the system performs in the fabrication of smart nanostructured materials.

**Results:**

We have here explored the nanoarchitecture and in vitro and in vivo functionalities of CXCR4-targeted, self-assembling protein nanoparticles intended for intracellular delivery of drugs and imaging agents in colorectal cancer. Interestingly, endotoxin-free materials exhibit a distinguishable architecture and altered size and target cell penetrability than counterparts produced in conventional *E. coli* strains. These variant nanoparticles show an eventual proper biodistribution and highly specific and exclusive accumulation in tumor upon administration in colorectal cancer mice models, indicating a convenient display and function of the tumor homing peptides and high particle stability under physiological conditions.

**Discussion:**

The observations made here support the emerging endotoxin-free *E. coli* system as a robust protein material producer but are also indicative of a particular conformational status and organization of either building blocks or oligomers. This appears to be promoted by multifactorial stress-inducing conditions upon engineering of the *E. coli* cell envelope, which impacts on the protein quality control of the cell factory.

## Background

The physiological diversity of microorganisms makes them suitable platforms for the cost-effective fabrication of a diversity of substances and macromolecules, including emerging nanostructured materials such as metal deposits, polymeric granules, functional amyloids and viruses or virus-like protein assemblies [1]. Being highly versatile, environmentally friendly and fully scalable, microbial fabrication is in many aspects more convenient than chemical synthesis [2–4]. Microbial cells, namely bacteria and yeast, have been exploited as factories for the production of many protein drugs that have been approved for use in humans. Historically, *Escherichia coli* was the pioneering cell factory for protein production because of the well-known genetics and metabolism. However, the presence of endotoxins in the cell envelope of Gram-negative bacteria poses an up to now unsolvable obstacle in the use of this organism for drug production [5]. Lipopolysaccharide (LPS) removal increases the complexity of protein purification processes, and it is recognized that LPS, even in low amounts, is a common contaminant of complex protein complexes such as bacteriophages [6] and also of plain recombinant proteins [7]. In fact, contaminant LPS and other cell envelope components are responsible for wrongly attributed biological effects of recombinant proteins tested in biomedical assays, and for poor biocompatibility (some examples can be found in [8–10]). In this context, Gram-positive bacteria, yeast and mammalian cells are endotoxin-free alternatives to *E. coli*, and their prevalence in protein drug production has steadily increased [11, 12], although not absent of problems.

Very recently [13], endotoxin-free *E. coli* strains have been developed by fine multi-step mutagenesis as a way to merge the versatility of *E. coli* as an advantageous protein production platform in the absence of endotoxic contaminants in its products. Those strains have been proved efficient in the production of diverse soluble protein species [13] and also of inclusion bodies intended as smart topologies [14] or as functional amyloids for intracellular protein release [15]. We wanted here to explore how the production, functionalities and nanoarchitecture of a complex, virus-like protein nanoparticle (T22-GFP-H6) designed as drug carrier [16] would be affected by the use of this particular fabrication platform. These particles are formed by a self-organizing, CXCR4-targeted polypeptide that accumulates, in the assembled form, in tumoral tissues of colorectal cancer animal models [16, 17]. Accumulation in tumor is promoted by the N-terminal peptide T22, which is a potent ligand of the cell surface cytokine receptor CXCR4 [16]. CXCR4 is overexpressed in colorectal cancer cells and linked to tumor aggressiveness [18]. The combination of the highly cationic T22 peptide with the C-terminal histidine tail in a modular protein promotes the self-assembly of the whole construct, favored by the bipolar charge distribution of the building block surface [19]. Recently, we have observed that strain-dependent genetic features of the producing bacteria impact on tumor targeting and on the whole-body biodistribution of the oligomeric construct upon its systemic administration [20]. Then, we were interested in dissecting the structure and activities of these tumor-homing nanoparticles when produced in endotoxin-free *E. coli* cells, whose complex genetic development might have resulted in relevant physiological shifts.

## Methods

### Strains, plasmids and media

T22-GFP-H6 is a tumor-targeted modular protein that self-assembles as toroid nanoparticles of about 12 nm of diameter. These oligomers are fully stable in vivo upon tail vein administration in colorectal cancer mice models [17] and accumulate intracellularly in primary tumor and in metastatic foci [16]. T22-GFP-H6 protein nanoparticles were produced in the KPM22 L11-derivative [21], endotoxin-free strain KPM335 (*msbA52*, Δ*gutQ*, Δ*kdsD*, Δ*lpxL*, Δ*lpxM*, Δ*pagP*, Δ*lpxP*, Δ*eptA*, *frr181*) and its parental K-12 strain BW30270 (CGSC#7925–MG1655; F^−^, *rph*^+^, *fnr*^+^) with a wild-type LPS. The design of KPM335, based on the incorporation of non-reverting deletions of seven genes to disrupt Kdo biosynthesis and modifications of lipid IV_A_, prevents the strain from regaining the potential to synthesize normal LPS or endotoxically active lipid IV_A_ derivatives through acquiring mutations. Also, *E. coli* K-12 MC4100 ([*araD139*], (*argF*-*lac*)169, λ—*relA1*, *rpsL150*, *rbsR22*, *flb5301*, *deoC1*, *pstF25* Strep^R^) and BL21 Origami B [F^−^*ompT hsdS*_*B*_(r_B_^−^ m_B_^−^) *gal dcm lacY1 ahpC* (DE3) *gor522*::Tn*10 trxB* (Kan^R^, Tet^R^)] (Novagen, Darmstadt, Germany) were used for protein production as controls. KPM335, BW30270 and MC4100 were transformed with plasmid pTrc99a, whereas Origami B was transformed with a pET22b-derived plasmid. All expression plasmids contained the T22-GFP-H6 DNA sequence optimized by the codon usage of *E. coli* and have been described previously [20]. Bacteria were always grown in Lysogeny Broth (LB) rich media [22]. Part of the protein was produced with the assistance of the Protein Production Platform of CIBER-BBN/IBB, at the UAB (http://www.nanbiosis.es/unit/u1-protein-production-platform-ppp/).

### Competent cells and transformation

Cultures were grown overnight at 37 °C with shaking at 250 rpm and used as a 1/100 inoculum in 50 ml of LB. After reaching an optical density (OD_550_) between 0.2 and 0.4, MC4100, BW30270 and Origami B cultures were centrifuged (4000×*g*) at 4 °C for 15 min. Pellets were resuspended in 12.5 ml of cold and sterile 50 mM CaCl_2_ and incubated for 45 min in an ice bath. Cells were centrifuged again as described above and resuspended in 1.25 ml of cold and sterile 50 mM CaCl_2_ in glycerol (15 % v/v) to prepare aliquots of 200 µl and stored at −80 °C. To transform the cells, 40 ng of plasmid DNA were added to the competent cells. The mixtures were incubated on ice for 30–60 min, warmed up to 42 °C for 45 s and placed on ice for 30 s. After incubation, 800 µl of LB media were added, and transformed cells were incubated at 37 °C for 1 h. Finally, the cells were plated on LB-agar plates containing the corresponding antibiotic. In the case of KMP335 strain, cells were grown to an OD_550_ between 0.2 and 0.4, placed on ice for 20 min and sedimented by centrifugation (3100×*g*, 4 °C, 20 min). The pellets were centrifuged and resuspended successively in 40, 20 and 10 ml of H_2_O (ice-cold and sterile), followed by a wash with 5 ml of 10 % glycerol (ice-cold and sterile). The final pellet was resuspended in 1 ml of 10 % glycerol (ice-cold and sterile) to prepare 50-µl aliquots for storage at −80 °C. Electrocompetent KPM335 cells were transformed by electroporation in pre-chilled 0.2 cm electroporation cuvettes using 50 µl of competent cells and 40 ng of plasmid DNA. Cells were pulsed using a Gene Pulser MX cell electroporator (Bio-Rad, Hercules, CA, USA) at 25 µF, 200 Ω, 2500 V and 4.7–4.8 ms. Immediately after the pulse, 800 µl of LB medium were added, and the mixture was incubated at 37 °C for 1 h. Cells were plated on LB-agar plates containing ampicillin (100 µg/ml) and incubated at 37 °C overnight.

### Protein production and purification

Overnight cultures were inoculated in 500 ml of LB media with 100 µg/ml ampicillin in 2-L flasks. Streptomycin (at 30 µg/ml) was also added to MC4100 cultures, and tetracycline (100 µg/ml) and kanamycin (33 µg/ml) to Origami B cultures. Each culture was grown at 37 °C and 250 rpm to an OD_550_ of about 0.5 before isopropyl-β-d-thiogalactoside (IPTG) was added to 1 mM to induce recombinant gene expression. T22-GFP-H6 was produced overnight at 20 °C with shaking (250 rpm) in all cases.

After overnight production, cultures were centrifuged (3280×*g*, 4 °C, 40 min), and the cell pellets were resuspended in 20 mM Tris–HCl, pH 8.0, containing 500 mM NaCl, 20 mM imidazole (buffer A), and an EDTA-free protease inhibitor cocktail (Complete EDTA-free, Roche Diagnostics, Indianapolis, IN, USA). Intra-cellular protein was extracted by cell disruption using a French press (Thermo FA-078A) at 1100 psi, and purified by His-tag affinity chromatography using a 1-ml HiTrap Chelating HP column (GE Healthcare, Piscataway, NJ, USA) in an ÄKTA purifier FPLC (GE Healthcare). Protein separation was achieved with a linear imidazole gradient from 20 mM to 500 mM, by diluting 20 mM Tris–HCl, pH 8.0, plus 500 mM NaCl and 500 mM imidazole (buffer B) in buffer A, and fractions were collected and dialyzed against 166 mM NaHCO_3_, pH 7.4. The amount of protein was determined by Bradford’s assay [23], and the integrity of the protein was analyzed by sodium dodecylsulfate polyacrylamide gel electrophoresis (SDS-PAGE), followed by Western blot analysis as described below.

### Western blot analysis

Separated fractions were subjected to 10 % SDS-PAGE according to Laemmli’s method [24]. Protein was transferred to nitrocellulose membranes (GE Healthcare, Piscataway, NJ, USA) that were blocked overnight with 5 % skim milk in PBS. After blocking, membranes were incubated with anti-GFP antibody (1:500, sc-8334, Santa Cruz Biotechnology, Santa Cruz, CA, USA), followed by incubation with a secondary HRP-conjugated anti-rabbit IgG (H + L) antibody (Bio-Rad, Hercules, CA, USA) at a dilution of 1:2000. Protein bands were visualized with a solution of 25 % cold methanol, 0.2 % H_2_O_2_ and 0.65 mg/ml of 4-chloronaftol in PBS, and images were obtained using a GS800 Calibrated Densitometer scanner (Bio-Rad).

### Fluorescence intensity

Fluorescence intensity of T22-GFP-H6 was determined in a Varian Cary Eclipse fluorescence spectrometer (Agilent Technologies, Mulgrave, Australia) at excitation and emission wavelengths of 450 and 510 nm, respectively.

### Particle size and zeta potential

Size distribution and zeta potential of nanoparticles were measured by dynamic light scattering (DLS) at 633 nm (Zetasizer Nano ZS, Malvern Instruments Limited, Malvern, Worcestershire, UK). Samples were analyzed by triplicate averaging of fifteen single measurements.

### Mass spectrometry (MS) analysis

Purified protein was diluted 1:2 with H_2_O MilliQ and dialyzed against 50 mM of NH_4_HCO_3_ for 1.5 h. After dialysis, protein was mixed with a matrix of 2.6 dihydroxyacetophenone (1:1), and 1 µl of the mixture was deposited on a ground steel plate. Samples were analyzed using a lineal method in an UltrafleXtreme MALDI-TOF instrument (Bruker Daltonics, Bremen, Germany) with ion acceleration of 25 kV.

### Circular dichroism (CD)

CD measurements were carried out in a Jasco J-715 spectropolarimeter at 25 °C and 10 µM protein in 160 mM NaHCO_3_, pH 7.4. CD and high-tension (HT) voltage spectra were obtained over a wavelength range of 200–250 nm at a scan rate of 50 nm/min, a response time of 0.5 s, and a bandwidth of 0.5 nm.

### Electron microscopy

To visualize the ultrastructure of protein nanoparticles by transmission electron microscopy (TEM), 2 µl of purified protein (0.2 mg/ml) were placed on carbon-coated copper grids for 1 min. Excess of sample was bloated and 2 µl of 1 % w/v uranyl acetate were added for negative staining. Samples were immediately visualized in a TEM Jeol JEM-1400 (Jeol Ltd., Tokyo, Japan) equipped with a Gatan CCD Erlangshen ES1000 W camera (Gatan Inc, Abingdon, UK) and operating at 80 kV. Ultrastructural analyses of nanoparticle morphology were complemented with imaging in a nearly native state with a Field Emission Scanning Electron Microscope (FESEM). For that, protein samples were directly deposited over silicon wafers (Ted Pella, Reading, CA, USA), air dried and observed with a high resolution *in*-*lens* secondary electron detector through a FESEM Zeiss Merlin (Zeiss, Oberkochen, Germany), operating at 2 kV.

### Analysis of cell internalization

HeLa cells were cultured at 37 °C in a humidified atmosphere with 5 % CO_2_ for 24 h and plated in treated 24 well plates (Nunclon surface, Nunc 150628) with MEM-α medium supplemented with 10 % fetal bovine serum (FBS) and 2 mM Glutamax (Gibco, Rockville, MD, USA). Later, medium was removed and the cells were washed with Dulbecco's phosphate-buffered saline (DPBS). Then, 25 nM of T22-GFP-H6 in 250 µl Optipro supplemented with 2 mM l-glutamine was added. After incubation at 37 °C for 1 h, cells were treated with trypsin in DPBS (1 mg/ml) for 15 min, centrifuged at 300×*g* for 15 min, and pellets were resuspended in 300 µl DPBS. Samples were analyzed by flow cytometry using a FACSCanto system (Becton–Dickinson, Franklin Lakes, NJ, USA) with a 15 W air-cooled argon-ion laser at 488 nm excitation for GFP.

### Confocal microscopy

HeLa cells were plated on a MatTek culture dish (MatTek Corporation, Ashland, MA, USA) at 150,000 cells/plate and incubated at 37 °C for 24 h. After incubation, medium was removed to wash the cells twice with DPBS. Optipro medium supplemented with 2 mM l-glutamine and 25 nM T22-GFP-H6 were added, and cells were incubated at 37 °C for 1 h. To visualize the samples, plasma membranes and the nucleus were labeled using 2.5 mg/ml CellMask Deep Red and 0.2 mg/ml Hoechst 33342 (Molecular Probes, Eugene, OR, USA), respectively. A confocal laser scanning microscope TCS-SP5 (Leica Microsystems, Heidelberg, Germany) was used to analyze the samples, and the images were processed through the Imaris Bitplane software (Bitplane, Zurich, Switzerland).

### Determination of in vivo biodistribution

This study was approved by the Institutional Animal Ethics Committee. We implanted CXCR4-overexpressing SP5 human colorectal tumor line to generate subcutaneous colorectal tumors in swiss nude mice (Charles River, France) as previously described [16, 17]. When tumors reached ca. 500 mm^3^, mice were randomly allocated to Origami B, MC4100, KPM335, BW30270 or buffer-treated groups (N = 3–5/group). The experimental mice received a single intravenous bolus of the corresponding nanoparticle (500 μg in carbonate buffer, pH 7.4), whereas control mice received only buffer. Five hours after the administration, we measured ex vivo the fluorescence emitted by the nanoparticles accumulated in the whole and slice sectioned tumor and normal tissues (kidney, lung, and heart, liver and brain) using IVIS^®^ Spectrum equipment (Perkin Elmer, Waltham, MA, USA). The fluorescence signal was digitalized, and after subtracting the autofluorescence, it was displayed as a pseudocolor overlay and expressed as Radiant efficiency. Data were corrected by the specific fluorescence emitted by the different nanoparticles. Signal difference between groups was determined by applying the non-parametric Mann–Whitney test.

### Histological and immunohistochemical evaluation

At necropsy, tumors were fixed with 4 % formaldehyde in PBS for 24 h and embedded in paraffin for histological and immunohistochemical evaluation. 4 µm sections, were processed as previously described [16, 17] and haematoxylin and eosin (H&E) stained for histological analysis, that was performed by two independent observers. We assessed CXCR4 membrane expression and nanoparticle cell internalization in tumor and normal tissues by IHC using primary anti-CXCR4 (1:300; Abcam, Cambridge, UK) or anti-GFP (1:100; Santa Cruz Biotechnology, CA, USA), and secondary HRP conjugated antibody, followed by chromogenic detection. We quantified the percentage of CXCR4-expressing cells in relation to the total cell number and their staining intensity, scoring each sample from 0 to 3 (where 3 is the maximal intensity) and multiplying both parameters to obtain its H-score. Representative pictures were taken using CellэB software (Olympus Soft Imaging v3.3, Tokyo, Japan) at 400×.

### Quantitative real-time PCR

For isolation of total RNA, triplicate cultures of *E. coli* strains BW30270 and KPM335 were grown aerobically with shaking (250 rpm) at 37 °C in LB medium to the mid-exponential growth phase at an OD_600_ of 0.6–0.7. The bacterial cells were harvested in the presence of RNAprotect Bacteria Reagent for immediate RNA stabilization prior to RNA isolation using the RNeasy Mini Kit and on-column DNase treatment in accordance with the recommendations of the manufacturer (Qiagen). RNA concentrations were determined using a NanoDrop 2000 spectrophotometer (Thermo Scientific), and the integrity of the RNAs was verified with RNA Nano Chips in an Agilent 2100 Bioanalyzer. The Maxima First Strand cDNA Synthesis Kit was used to generate cDNA from 0.3 µg RNA following the manufacturer´s instructions (Thermo Scientific, Waltham, MA, USA). The cDNA levels of chaperone genes were then analyzed by quantitative real-time PCR (RT-qPCR) using a LightCycler 480 Instrument II with LightCycler 480 SYBR Green I Master reaction mixes according to the instructions of the manufacturer (Roche Diagnostics). RT-qPCR was performed with an initial denaturation at 95 °C for 5 min, followed by 45 cycles of 10 s at 95 °C, 10 s at 60 °C, and 10 s at 72 °C. Each sample was analyzed in duplicate using 1:15 and 1:50 dilutions of the cDNAs and the gene-specific primers listed in Table 1. The primer pairs (Table 1) were designed following the rules of highest possible sensitivity and efficiency with the Primer3 2.3.4 plugin of the Geneious v9.0.5 software (Biomatters, Auckland, New Zealand). Relative expression levels of the target genes were normalized to the internal reference gene *ihfB* for the β-subunit of the integration host factor using the comparative 2^−∆∆Ct^ method [25].

**Table 1:** Primers used for RT-qPCR

Gene	Primer	Sequence	Amplicon length (bp)
*tig*	ECtig353F	AGGGTCTGGAAGCGATCGAAG	301
	ECtig653R	GGGAAGGTCACGTCGATGGT	
*ibpA*	ECibpA85F	CAGAGTAATGGCGGCTACCCT	299
	ECibpA383R	GCTTCCGGAATCACGCGTTC	
*hscB*	EChscB191F	CGTTAATGCGCGCGGAATATTTG	301
	EChscB491R	AGTTGTTCGGCACTGCTTCG	
*hchA*	EChchA398F	TCGCGGATGTTGTTGCCAG	300
	EChchA697R	CGCCGAAGTACCAGGTGAGA	
*grpE*	ECgrpE278F	ACGAATTGCTGCCGGTGATTG	301
	ECgrpE578R	GCTACAGTAACCATCGCCGC	
*groL*	ECgroL583F	TTCGACCGTGGCTACCTGTC	301
	ECgroL883R	GGGTTGCGATATCCTGCAGC	
*dnaK*	ECdnaK504F	CATCAACGAACCGACCGCAG	300
	ECdnaK803R	TTTTCTGCCGCTTCTTTCAGGC	
*clpB*	ECclpB426F	AATGCGTGGAGGTGAAAGCG	300
	ECclpB725R	ATATCCAGCGCCAGTACCCG	
*clpA*	ECclpA287F	AGTCCTCCGGTCGCAATGAG	300
	ECclpA586R	CCAGCTCCTTCTCACGACCA	
*cbpA*	ECcbpA477F	GCTGAATGTGAAGATCCCGGC	300
	ECcbpA776R	ACCAGACCTTTGCCTTTAACGC	
*ihfB* ^a^	ECihfB9F	GTCAGAATTGATAGAAAGACTTGCCACC	258
	ECihfB266R	CGATCGCGCAGTTCTTTACCAG	

^a^ Used for normalization of gene expression

## Results and discussion

**Fig. 1:**
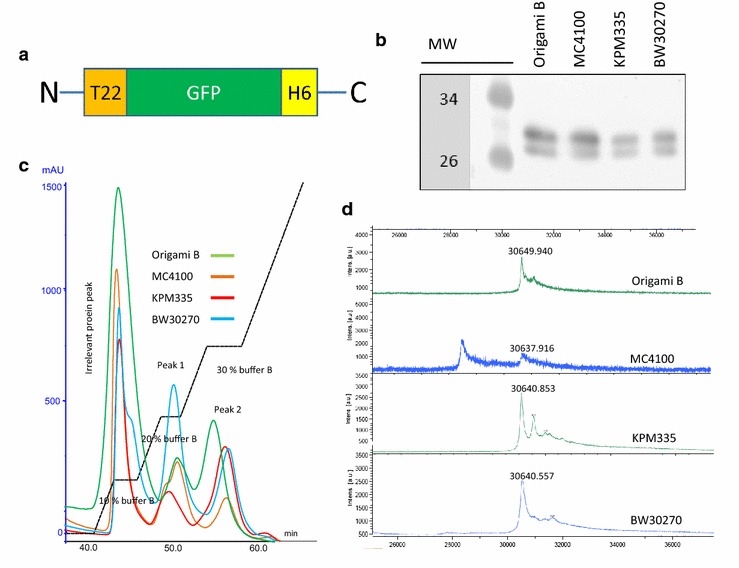
Protein production and purification. **a** Scheme of the modular T22-GFP-H6 building block. Sizes of boxes are only approximate and do not precisely correspond to the length of primary aa sequence. **b** Western blot of crude cell extracts of T22-GFP-H6-producing strains. MW indicates the molecular weight of selected markers. **c** His-tag affinity chromatography of T22-GFP-H6 produced in the different *E. coli* strains. Purification was performed using an imidazole concentration gradient. Buffer B contains 500 mM imidazole. **d** Maldi-TOF identification of T22-GFP-H6 produced in different *E. coli* strains and purified by affinity chromatography (P2)

**Fig. 2:**
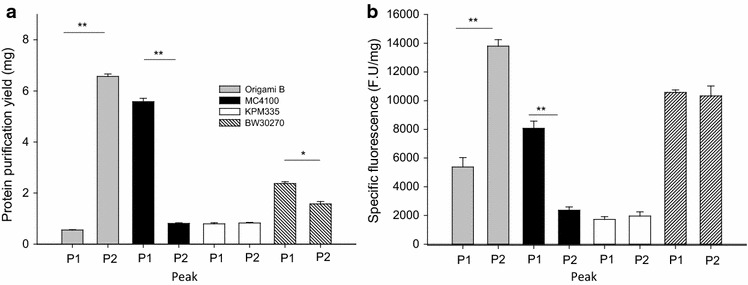
Quantitative analyses of T22-GFP-H6 production levels and activity. **a** Yield of T22-GFP-H6 produced in *E. coli* strains Origami B, MC4100, KPM335 and BW30270, upon purification, from 500 ml of original protein producing cultures. *P1* and *P2* indicate the main elution peaks 1 and 2. **b** Specific fluorescence of T22-GFP-H6 calculated by using the above data. Symbols mean significant differences: **p < 0.001; *p < 0.05

The self-assembling protein T22-GFP-H6 (Fig. 1a) was produced in the endotoxin-free strain KPM335 and its parental BW30270, as well as in MC4100 and Origami B strains, used in previous studies for the routine fabrication of T22-GFP-H6 nanoparticles. While the generation time of KPM335 has increased by about 50 % in comparison to the parental wild-type strain, no signs of instability, lysis, foaming or cell death could be observed. In all cases, T22-GFP-H6 appeared as a stable full-length product showing the expected molecular mass of about 30 kDa (Fig. 1b). The steadily observed double band was probably due to different conformational species of the protein. When purified by His affinity chromatography from bacterial cell extracts, T22-GFP-H6 was eluted in two separated peaks (P1 and P2) irrespective of the strain used for production (Fig. 1c). P2 has been previously observed as the one containing protein materials more efficient in cell penetration assays [20]. MS of these P2 samples revealed the expected molecular masses of all protein variants with minor signs of proteolytic instability (more prevalent in MC4100 through the occurrence of a degradation fragment of 28.4 kDa; Fig. 1d), also confirming that the double band observed in blots (Fig. 1b) was indeed due to conformational variability instead to significant occurrence of truncated forms. Both protein yield and emitted fluorescence were significantly lower in KPM335 than in other strains (Fig. 2a), as well as the specific fluorescence emission of T22-GFP-H6 (Fig. 2b). While a moderate protein yield in recombinant KPM335 was not unexpected according to previous reports referring to different produced protein species [13], a specific fluorescence emission lower than that observed in its parental BW30270 (and in other reference strains) was indicative of a differential conformation of T22-GFP-H6 monomers or an alternate configuration of the resulting nanoparticles.

**Fig. 3:**
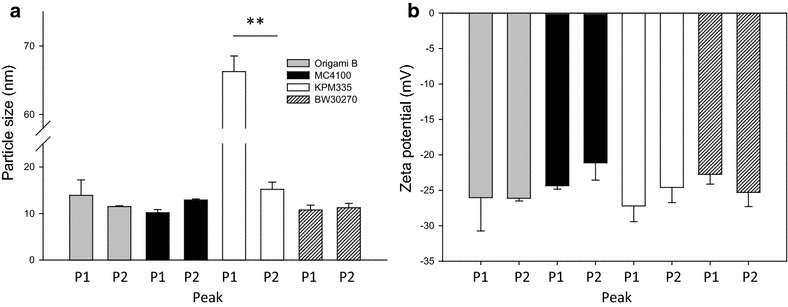
Physical properties of protein nanoparticles. **a** Size of the self-assembled nanoparticles purified in two elution peaks and measured by DLS. **b** Surface charge distribution of self-assembled nanoparticles measured as zeta potential. Symbols mean significant differences: **p < 0.001; *p < 0.05

**Fig. 4:**
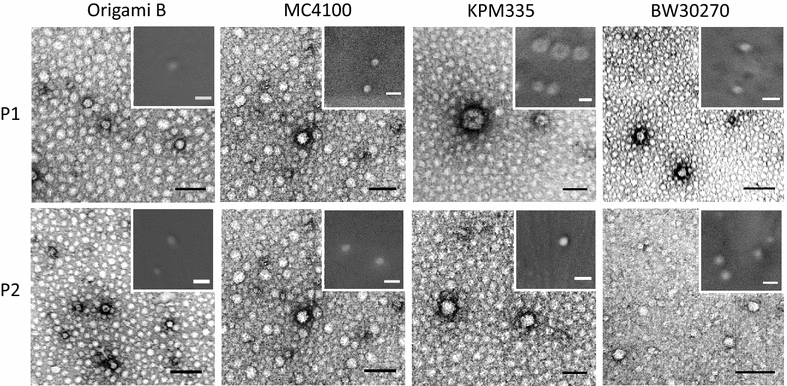
Morphometric characterization of nanoparticles ultrastructure. Representative TEM and FESEM (insets) images of all strains and main elution peaks 1 and 2. *Scale bars* represent 50 nm

The formation of nanoparticles was firstly confirmed by DLS, revealing a regular size of the material ranging between 10 and 15 nm, depending on the cell factory (Fig. 3a). Interestingly, and according to the above hypothesis of a particular organization of T22-GFP-H6 as produced in KPM335, P1 nanoparticles produced in this strain were particularly large, reaching around 75 nm in diameter (Fig. 3a). This variability did not result in appreciable modifications in the superficial charge of protein nanoparticles (Fig. 3b). The observed deviations in the size of protein materials were fully confirmed by TEM and FESEM morphometric analyses (Fig. 4).

**Fig. 5:**
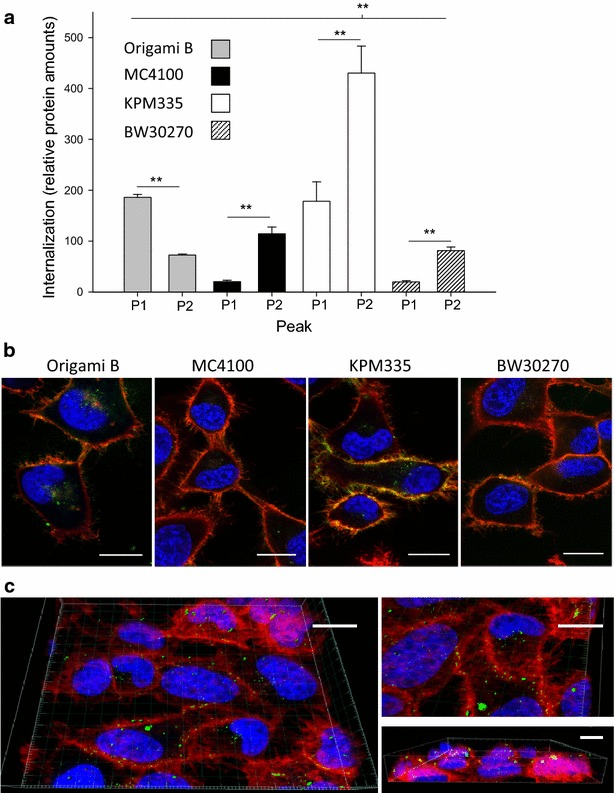
Cell penetrability of nanoparticles. **a** Internalization of self-assembling nanoparticles analyzed by intracellular fluorescence of HeLa cells incubated with 25 nM of T22-GFP-H6 for 1 h. Internalize fluorescence (determined by flow cytometry) is corrected by specific fluorescence (determined by fluorimetry). The *quotient* represents relative protein amounts. **b** Internalization of nanoparticles visualized by confocal microscopy. Nucleus (*blue*) of HeLa cells were labeled using Hoechst 33342, membranes (*red*) were labeled with CellMask Deep Red. Cells were incubated with 25 nM of T22-GFP-H6 (*green*) for 1 h. *Bars* indicate 15 µm. **c** Three dimensional 3D reconstruction of T22-GFP-H6 nanoparticles (P2) from KPM335 into HeLa cells. Images were obtained using 35 layers per image. Three different fields of confocal microscopy as well as three angles were viewed through Imaris Bitplane Software. *Scale bars* indicate 15 µm and symbols mean significant differences: **p < 0.001; *p < 0.05

When analyzing penetrability into CXCR4^+^ HeLa cells in culture, a convenient in vitro model for T22-empowered nanoparticles [16], P2 materials resulted generically more efficient than P1’s (Fig. 5a). The exception found in Origami B cells is in agreement with previous observations [20], and it might be related to the less reducing cytoplasm in this bacterial strain and caused by a bottom-up impact of protein conformation on microscopic functioning of the particles in cell interfaces, associated to favored di-sulfide bridge formation in this factory. Cell penetrability of P2 particles was comparatively screened again by confocal microscopy and the enhanced performance of those produced in KPM335 fully confirmed (Fig. 5b). These nanoparticles accumulated intracellularly in the perinuclear region, showing moderate nuclear penetrability (Fig. 5c).

**Fig. 6:**
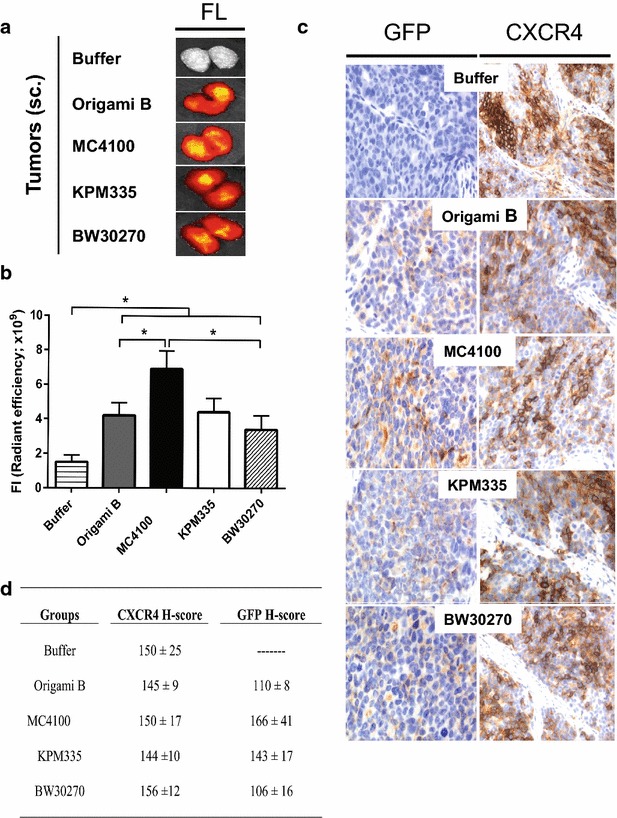
In vivo biodistribution of nanoparticles in tumor tissue. **a** Protein accumulation determined by ex vivo imaging of GFP-emitted fluorescence by representative subcutaneous colorectal cancer tumors grown in mice (measurements performed 5 h after the intravenous administration of a 500 μg nanoparticle dose). **b** Quantitation of GFP-emitted fluorescence in tumors of the compared groups, expressed as total radiant efficiency (photon/s/cm^2^/sr/μW/cm^2^). **c** Representative micrographs of nanoparticle internalization, as detected by IHC with an anti-GFP antibody, in the tumor cell cytosol and CXCR4 expression observed in tumor cells before nanoparticle injection in representative tumors. **d** Mean ± S.E. of CXCR4 expression H-score in tumor tissue (measured prior to nanoparticle administration) and GFP H-score (a measure of nanoparticle internalization in tumor cells) of mice belonging to the Origami B, MC4100, KPM335, BW30270 or buffer-treated groups. Note the similar level of CXCR4 expression in tumors among groups and the correlation between tumor-emitted fluorescence and the amount of nanoparticle internalized in tumor cells

**Fig. 7:**
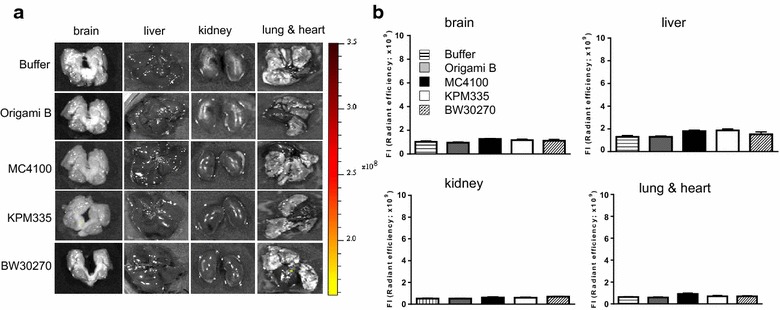
Nanoparticle biodistribution in normal tissues. **a** Representative ex vivo images of T22-GFP-H6 nanoparticles uptake in mouse brain, liver, kidney, lung or heart tissue after injection as measured by GFP-emitted fluorescence. **b** Quantitation of GFP fluorescence expressed as radiant efficiency. Note the negligible level of detected fluorescence, being undistinguishable from the background fluorescence observed in vehicle-treated mice. This was in contrast to the high level of nanoparticle accumulation observed in tumor tissues in the same animals (see Fig. 6)

The performance of P2 nanoparticles, those showing higher cell penetrability in vitro, was evaluated in vivo through analyzing their biodistribution and accumulation in CXCR4-overexpressing tumor tissues, in a subcutaneous mouse model of colorectal cancer. Upon systemic administration, all protein variants targeted and accumulated in the primary tumor, albeit to different extents (Fig. 6a–c), with materials produced in MC4100 being the most efficient. Nanoparticles produced in KPM335 showed results similar in efficacy to those manufactured in Origami B, the paradigmatic cell factory for T22-GFP-H6 [17], and slightly better than the parental BW30270 (although differences were not significant). In any case, the higher cell penetrability of these nanoparticles determined in cultured cell (Fig. 5a) was not reflected by an enhanced tumor targeting in vivo. As expected, accumulation of protein nanoparticles was coincident with the overexpression of CXCR4 in target tissues (Fig. 6d, e), and none of the protein materials was observed to accumulate in the evaluated normal non-target organs (namely kidney, lung, heart, liver, spleen and pancreas; Fig. 7a, b). The absence of a signal in kidney was indicative of a high stability in vivo of the nanoparticles that keep assembled in oligomers of size over the cut-off of renal clearance (~8 nm).

**Fig. 8:**
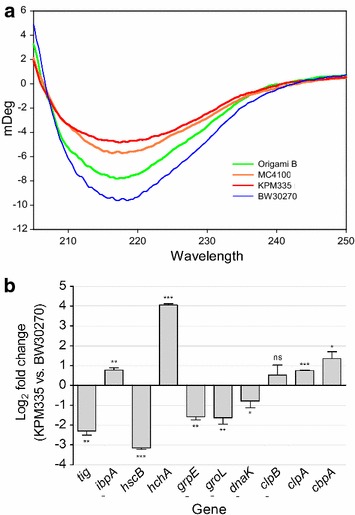
Conformational protein status and activity of the cell’s quality control. **a** Smoothed CD spectra from 250 to 205 nm of T22-GFP-H6 produced by different *E. coli* strains and purified at higher imidazol concentration (P2). Beta sheet structure signal is detected at 218 nm. **b** Expression of chaperone genes in *E. coli* KPM335 compared to their expression levels in *E. coli* BW30270. Relative changes in expression of target genes as quantified by RT-qPCR using the comparative 2^−∆∆Ct^ method [25] and the *ihfB* gene for normalization were converted into log2 values to display the fold changes in log2 scale. The values represent the means and standard deviations based on duplicate RT-qPCR runs for each of the three independent biological replicates per strain. The statistical significance of the differential expression patterns was analyzed using the paired t test. *P < 0.05; **P < 0.01; ***P < 0.001; ns, not significant. Genes labelled with a dash are those whose expression change (either up- or down-regulation) is coincident with the proteomic analysis of a conventional *E. coli* strain when entering into the stationary phase (according to data from [26]). The *tig, hscB, hchA* and *cbpA* gene products were not monitored in this previous study

Although the final performance of KPM335 materials in vivo was excellent and indistinguishable from that of Origami B-derived nanoparticles, the larger nanoparticle size observed in the P1 fraction (Figs. 3a, 4), and particularly the higher cell penetrability of the P2 fraction in vitro were indicative of either a different conformation of T22-GFP-H6 building blocks, a diversity in the architecture of nanoparticles produced in this particular strain, or both. Conformational protein variations were indeed identified by CD of P2 protein fractions (Fig. 8a), distinguishing KPM335- and BW30270-derived materials as the extremes of a conformational spectrum. Since BW30270 is the endotoxin-containing, parental strain of the endotoxin-free KPM335 [13], the results suggest that genetic tailoring associated with LPS modification might have affected cell properties related to protein metabolism.

In a previous study [20], we have demonstrated that mutations in the *E. coli* genome that affect protein quality control activities (chaperones and proteases) impact on the architecture and function of produced protein nanoparticles [20]. Also, the growth of cultured KPM335 is slightly slower than that of the parental strain BW30270 [15]. Reduced bacterial growth, as promoted, for instance, by minimal defined media or by entering into the stationary phase, results in the modification of intracellular levels of chaperones such as increased levels of ClpB and IbpA and reduced levels of GrpE, HtpG and Hsc [26]. In this context, we wondered if the LPS engineering of KPM335 might have resulted in a physiological, indirect impact on protein processing by a modified cell factory through modification of chaperone balance. Transcriptomic analysis of main folding-assistant genes in KPM335 revealed significant differences regarding the parental BW30270 (Fig. 8b), and in some cases a pattern of expression that might be comparable to that found in proteomics of wild-type bacteria at the end of the exponential growth phase (Fig. 8b). In particular, *tig* transcript levels are lower being the production of trigger factor associated with ribosomal protein expression and growth rate [26]. On the other hand, some heat shock protein genes are upregulated, in particular those involved in inclusion body disaggregation (c*lpB* and *ibpA*) [27, 28] indicating enhanced aggregation in the endotoxin-free strain when comparing with the parental background. In this regard, the formation of inclusion bodies by an aggregation-prone protein is slightly favoured in KPM335 when compared to BW30270, as measured by the percentage of insoluble recombinant protein over the total protein yield (86.7 ± 1.2 versus 81.649 ± 1.4 %; data calculated from [15]). Also, ClpB and IbpA also respond, independently of protein production, to growth rate. They are present in higher amounts in stationary phase at least when cells are growing in complex medium and the growth rate declines gradually [26]. On the other hand, some heat shock protein genes are downregulated (e.g. *grpE*), that is again in agreement with the endotoxin-free strain growing slower, as there is a slight tendency that these heat shock proteins do not change with growth rate or they are present in lower amounts when the cells grow slower or approach stationary phase [26].

## Conclusions

In summary, the set of seven different mutations in LPS biosynthesis genes of KPM335 promotes a reduction of the cell growth rate that impact, intrinsically, on the expression of several among the main chaperones of the *E. coli* quality control network. This may have indirect influences on the conformational status of recombinant aggregation-prone protein materials and their building blocks. Although a more complete systems level analysis of KPM335 performance as a cell factory would be desirable, the set of data presented here indicates that LPS engineering secondarily affects the arm of the cells' biosynthetic and maintenance machineries dealing with protein conformation. While this fact does not negatively affect the final quality (tumor-homing) and organization (self-assembling) of complex smart materials such as CXCR4-targeted protein nanoparticles, it indeed influences some of their relevant properties at the macroscopic level.
